# Anti-Angiogenic and Anti-Scarring Dual Action of an Anti-Fibroblast Growth Factor 2 Aptamer in Animal Models of Retinal Disease

**DOI:** 10.1016/j.omtn.2019.07.018

**Published:** 2019-08-01

**Authors:** Yusaku Matsuda, Yosuke Nonaka, Satoshi Futakawa, Hirotaka Imai, Kazumasa Akita, Toshiaki Nishihata, Masatoshi Fujiwara, Yusuf Ali, Robert B. Bhisitkul, Yoshikazu Nakamura

**Affiliations:** 1 RIBOMIC, Inc., 3-16-13 Shirokanedai, Minato-ku, Tokyo 108-0071, Japan; 2 Department of Ophthalmology, University of California, San Francisco, San Francisco, CA, USA; 3 Institute of Medical Science, The University of Tokyo, Minato-ku, Tokyo 108-8639, Japan

**Keywords:** FGF2, aptamer, AMD, VEGF inhibitors, angiogenesis, fibrosis

## Abstract

Currently approved therapies for age-related macular degeneration (AMD) are inhibitors against vascular endothelial growth factor (VEGF), which is a major contributor to the pathogenesis of neovascular AMD (nAMD). Intravitreal injections of anti-VEGF drugs have shown dramatic visual benefits for AMD patients. However, a significant portion of AMD patients exhibit an incomplete response to therapy and, over the extended management course, can lose vision, with the formation of submacular fibrosis as one risk factor. We investigated a novel target for AMD treatments, fibroblast growth factor 2 (FGF2), which has been implicated in the pathophysiology of both angiogenesis and fibrosis in a variety of tissue and organ systems. The anti-FGF2 aptamer, RBM-007, was examined for treatment of nAMD in animal models. In *in vivo* studies conducted in mice and rats, RBM-007 was able to inhibit FGF2-induced angiogenesis, laser-induced choroidal neovascularization (CNV), and CNV with fibrosis. Pharmacokinetic studies of RBM-007 in the rabbit vitreous revealed high and relatively long-lasting profiles that are superior to other approved anti-VEGF drugs. The anti-angiogenic and anti-scarring dual action of RBM-007 holds promise as an additive or alternative therapy to anti-VEGF treatments for nAMD.

## Introduction

Age-related macular degeneration (AMD) is a leading cause of blindness in the developed world, with 10% to 20% of adults older than 65 years at risk and approximately 8 million people affected in the United States.^1^, ^2^ The annual incidence (estimated from prevalence) of late AMD in American whites was 3.5 per 1,000 aged 50 years or older, equivalent to 293,000 new cases in American whites per year.^3^ Incidence rates approximately quadrupled per decade in age.^3^ Neovascular AMD (nAMD, often referred to as “wet” AMD) is associated with the onset of choroidal neovascularization, which produces acute loss of central visual function because of vascular leakage and exudation, subretinal hemorrhage, and eventual subretinal fibrosis. The prevalence of nAMD in American whites is 0.84%, about one-tenth of non-nAMD (often referred to as “dry” AMD).^4^ VEGF, a potent endothelial cell mitogen and vascular permeability factor, is a major contributor to the pathogenesis of nAMD. VEGF is secreted by retinal pigment epithelial (RPE) cells from the basal side, directing paracrine stimulation toward choroidal blood vessels, which express high levels of VEGF receptors,^5^, ^6^ resulting in new blood vessels that originate from the choroid, break through Bruch’s membrane, and infiltrate the macula.^7^

Current US Food and Drug Administration (FDA)-approved therapies for nAMD are pegaptanib (Macugen, Eyetech Pharmaceuticals, New York, NY, USA), ranibizumab (Lucentis, Roche-Genentech, South San Francisco, CA, USA), and aflibercept (Eylea, Regeneron Pharmaceuticals, Tarrytown, NY, USA). Also used off-label is aliquoted bevacizumab (Avastin, Roche-Genentech, South San Francisco, CA, USA).^2^, ^6^ All of these agents act against the same target, vascular endothelial growth factor (VEGF). Treatments with anti-VEGF drugs, which are delivered by frequent intravitreal injections, have shown dramatic visual benefits for AMD patients.^7^, ^8^, ^9^ However, there are some critical limitations: in clinical trials, 23% of eyes treated monthly with ranibizumab proceeded to vision worse than 20/200, and 20% to 40% failed to resolve macular fluid even after 2 years of therapy.^7^, ^8^ Furthermore, in the “real world” setting, patients receive intravitreal injections at a much lower frequency than in trial protocols so that, on average, long-term visual outcomes with AMD treatment are poor.^10^, ^11^, ^12^ Factors associated with poor vision outcomes, in addition to persistent exudation, include macular atrophy and submacular fibrotic scar formation.^13^ Thus, there is a need for additive or alternative therapy to anti-VEGF treatments for nAMD.

Fibrosis is a common response of a tissue to injury from mechanical, inflammatory, ischemic, or degenerative causes.^14^ Subretinal fibrosis often develops in the natural progression of nAMD and can be associated with profound vision loss as choroidal neovascularization (CNV) progresses from a neovascular membrane to a variably mixed fibrovascular structure and eventually culminates in a scar, causing local destruction of photoreceptors and RPE.^15^, ^16^, ^17^, ^18^, ^19^ Submacular fibrosis can develop despite anti-VEGF treatment and has been identified as a cause of poor therapeutic outcomes in nAMD.^20^ The factors associated with scarring after anti-VEGF therapy have not been clarified.

**Figure 1:**
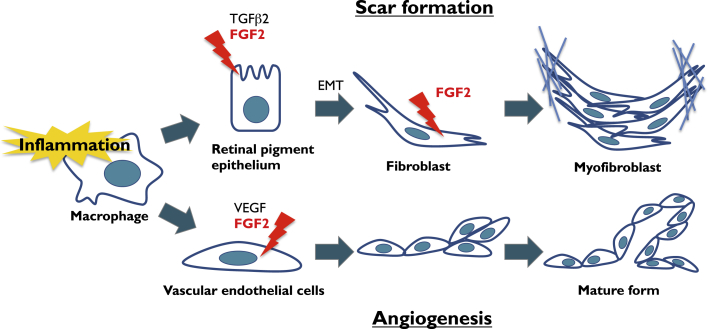
FGF2 in Angiogenesis and Fibrotic Scar Formation Shown is a schematic model of the dual activities of fibroblast growth factor 2 (FGF2) in the retina. Triggered by inflammation, FGF2 in the presence of transforming growth factor β2 (TGF-β2) stimulates retinal pigment endothelial cells to undergo epithelial-mesenchymal transformation (EMT) to fibroblasts in scar formation. In parallel, FGF2 acts as a vascular endothelial cell mitogen in the initiation and maturation of angiogenic vessels.

Fibroblast growth factor (FGF) has been implicated in the pathophysiology of both angiogenesis and fibrosis in several diseases (Figure 1).^21^, ^22^ FGF comprises a family of 22 cell-signaling proteins that act through four types of FGF receptor tyrosine kinases and have broad effects on multiple cell types during development, tissue remodeling and repair, and tumor formation.^23^, ^24^ In the 1980s, FGF1 and FGF2 were identified as angiogenic factors even prior to VEGF.^24^ An intimate cross-talk exists among FGF2 and the different members of the VEGF family during angiogenesis, lymphangiogenesis, and vasculogenesis.^25^ FGF2 promotes growth of vascular endothelial cells and tubular structure formation^26^ and stimulates VEGF production.^27^, ^28^ The mouse corneal micropocket assay has revealed that the angiogenic activity of FGF2 is stronger than that of VEGF.^29^, ^30^ However, the role of FGF2 in progression of nAMD has never been investigated.

Similarly, although FGF2 is characterized by stimulating fibroblast proliferation, the role of FGF2 in fibrotic scar formation in the retina has not been investigated. FGF2 is known to induce epithelial-mesenchymal transformation (EMT), in conjunction with transforming growth factor β (TGF-β) isomers, for organ remodeling during fibrogenesis. For example, FGF2 and TGF-β1 induce renal tubulointerstitial scarring by facilitating EMT in tubular epithelial cells.^31^ Likewise, in tooth development, FGF2 and TGF-β1 stimulate the EMT of Hertwig’s epithelial root sheath cells for cementum formation.^32^ There are some reports that, in the eye, TGF-β2, not TGF-β1, plays a role in scarring associated with eye pathologies, including proliferative vitreoretinopathy.^33^, ^34^, ^35^, ^36^ However, to our knowledge, there have been no reports to date of FGF2 in retinal fibrosis via EMT in RPE cells.

Here we investigated the effects of FGF2 inhibition via a neutralizing aptamer, RBM-007 (formerly called APT-F2P)^37^ *in vitro* and in animal models of CNV and subretinal fibrosis. An aptamer is a short single-stranded nucleic acid molecule that is selected *in vitro* from a large random sequence library by a process known as SELEX (systematic evolution of ligands by exponential enrichment).^38^, ^39^ The concept is based on the ability of short oligonucleotides to fold, in the presence of a target, into unique three-dimensional structures that bind the target with high affinity and specificity. RBM-007 is composed of 37 nt with a strong affinity for FGF2 (K_D_ < 20 pM),^37^ whose ribose 2′ positions are heavily modified to resist ribonucleases, and conjugated with 40-kDa polyethylene glycol (PEG), achieving a good pharmacokinetic profile (T_1/2_ > 24 h). Previous studies have shown that RBM-007 is able to block FGF2-induced signaling under equimolar conditions in several cell lines *in vitro* and to significantly reduce the severity of bone destruction affected by excess FGF2 in animal models.^37^

## Results

### Profiles of RBM-007

RBM-007 is an anti-FGF2 aptamer composed of 37 nt. Its ribose 2′ positions are heavily modified to resist ribonucleases, and its 5′ and 3′ termini are conjugated with 40-kDa PEG and an inverted deoxythymidine (dT), respectively, to achieve sufficient pharmacokinetics profiles.^37^ It has been shown that RBM-007 binds stably and specifically to FGF2 but no other FGF family proteins or heparin-binding proteins.^37^ Importantly, RBM-007 blocks binding of human FGF2 to its human receptors FGFR1–FGFR4.^37^ Furthermore, RBM-007 also blocks binding of murine FGF2 to its receptors.^37^ In this study, we determined the K_D_ of the non-PEGylated form of RBM-007 to FGF2 proteins from different species such as human, rat, mouse, and rabbit by a surface plasmon resonance (SPR) assay using a streptavidin sensor chip on which 5′-biotin-labeled RBM-007 oligonucleotide was immobilized. Sensorgrams after injection of different concentrations of FGF2 proteins were analyzed, and kinetic parameters were estimated (Figure S1). The K_D_ values ranged between 2 pM and 27 pM, indicating a high affinity of RBM-007 for different FGF2s beyond the species difference.

### The Involvement of FGF2 in EMT of RPE Cells

We assessed the effects of FGF2 and RBM-007 on EMT in RPE cells in the presence of TGF-β2. Prior to this combination treatment, RPE cells cultured at 37°C were treated individually with TGF-β2 (3 ng/mL) and FGF2 (1, 10, and 100 ng/mL), and the mRNA expression levels of the EMT biomarker α-smooth muscle actin (SMA; a component of myofibroblasts) and/or collagen type I (a product derived from myofibroblasts) were evaluated with quantitative real-time PCR. As reported in the literature, TGF-β2 alone induced EMT, whereas FGF2 alone in three doses failed to induce EMT (Figure S2). EMT of RPE cells was also confirmed by morphology changes induced by TGF-β2 alone or TGF-β2 plus FGF2. The number of spindle-shaped (fibroblast-like) cells increased upon treatment with TGF-β2 and increased further with TGF-β2 plus FGF2 (Figure S3).

**Figure 2:**
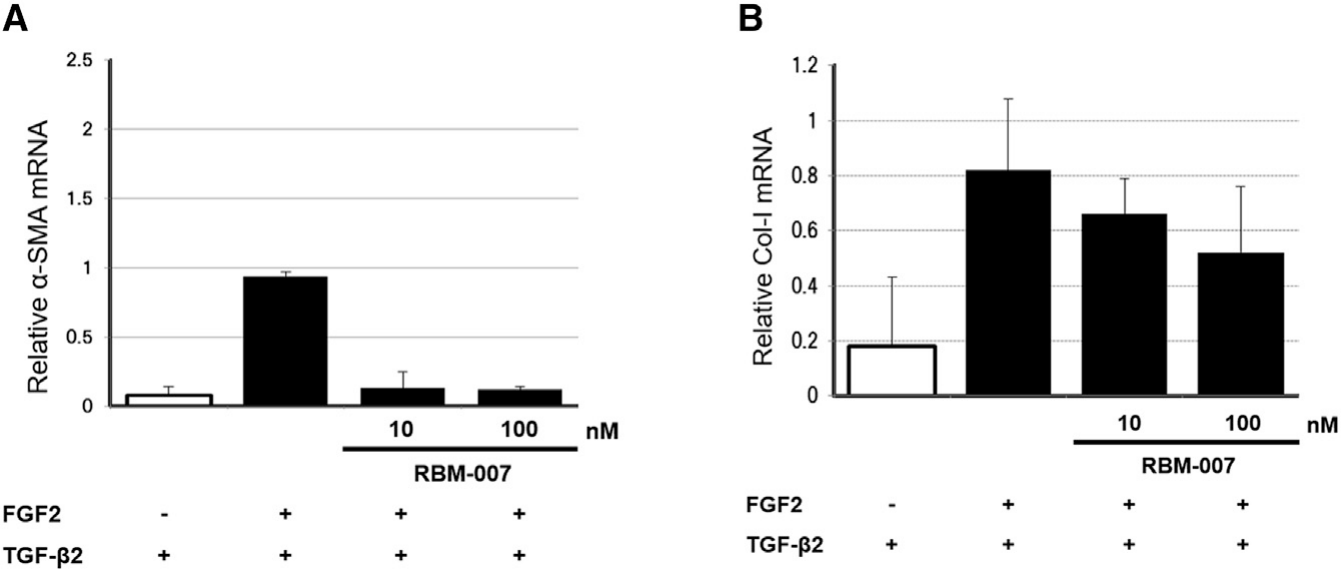
Attenuation of FGF2/TGFβ2-induced EMT in RPE cells by RBM-007 (A and B) The mRNA levels of α-SMA (A) and collagen type I (B) were examined by quantitative real-time PCR amplification in RPE cells after FGF2 and TGF-β2 stimulation in the presence or absence of RBM-007. Values are the mean and SD from three independent experiments. Note the indicated doses for 72 h.

Then RPE cells cultured at 37°C were treated with FGF2 (2 ng/mL) and TGF-β2 (3 ng/mL) with and without RBM-007 (10 and 100 ng/mL), and the mRNA expression levels of α-SMA and collagen type I were evaluated by quantitative real-time PCR. The results indicated that, in the presence of TGF-β2, FGF2 markedly stimulates expression of α-SMA, and this upregulation is blocked in a dose-dependent manner by RBM-007 (Figure 2; p < 0.05). A similar tendency was observed for expression of collagen type I, although the statistical significance was not confirmed. These findings reveal that FGF2, in concert with TGF-β2, triggers EMT in RPE cells as a co-facilitator^31^ and that RBM-007 inhibits EMT of FGF2-stimulated RPE cells.

### Effects of RBM-007 on Angiogenesis Activity in an FGF2-Induced Angiogenesis Mouse Model

**Figure 3:**
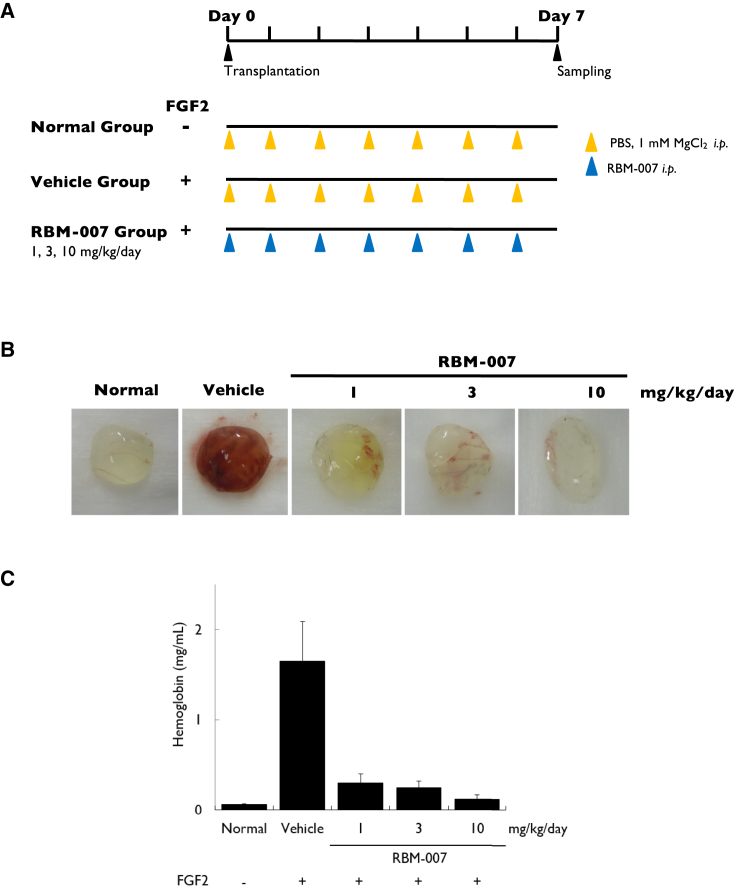
Matrigel Plug Assay of Angiogenesis in Mice (A) Experimental protocol. (B) Matrigel plug color, showing FGF2-stimulated blood vessel formation in the presence or absence of RBM-007 on day 7. (C) Hemoglobin levels in Matrigel plugs were measured, showing a dose-related reduction with daily intraperitoneal RBM-007 injection (p = 0.12). Values are the mean and SE of three or four independent experiments. Statistical analysis was performed using Steel’s test (non-parametric test) because the p value of Bartlett’s test was less than 0.05.

We examined the activity of RBM-007 to block FGF2-induced angiogenesis in mice using the Matrigel plug assay. Matrigel carrying FGF2 (1 μg) was subcutaneously implanted into mice, and RBM-007 was administered intraperitoneally every day at doses of 1, 3, and 10 mg/kg. Matrigel plugs were removed and photographed on day 7 post-implantation (Figures 3A and 3B). In controls, FGF2 stimulated new vessel formation in the Matrigel plugs, which was reduced by RBM-007 treatment at all doses (Figure 3B). Additionally, when the hemoglobin concentration in Matrigel plugs was measured, they were greatly increased by addition of FGF2 (mean 1.65 mg/mL 0.44 SE versus mean 0.06 mg/mL ± 0.01 SE for controls); a significant reduction was seen in groups treated with RBM-007 (0.30 ± 0.10, 0.25 ± 0.07, and 0.12 ± 0.05 for RBM-007 doses of 1, 3, and 10 mg/kg/day, respectively) (Figure 3C). Daily intraperitoneal administration of RBM-007, even at the lowest 1 mg/kg dose, was sufficient to inhibit FGF2-induced angiogenesis.

### Neovascularization-Inhibitory Effect of RBM-007 in a Mouse Model of Laser-Induced CNV

To evaluate the CNV-inhibitory effect of RBM-007, *in vivo* studies were conducted in a mouse model of laser-induced CNV in male C57BL/6J mice (6 weeks, n = 8∼10 per dose group). Immediately after completion of 6 laser burns in the retina-choroid, eyes received intravitreal injection of (1) saline vehicle, (2) ranibizumab, (3) RBM-007, or (4) a combination of ranibizumab and RBM-007. Seven days after laser irradiation, a 4% fluorescein isothiocyanate (FITC)-dextran solution was administered into the tail vein prior to sacrifice, enucleation, and globe fixation to prepare choroidal flat mounts for assessment by confocal microscopy.

**Figure 4:**
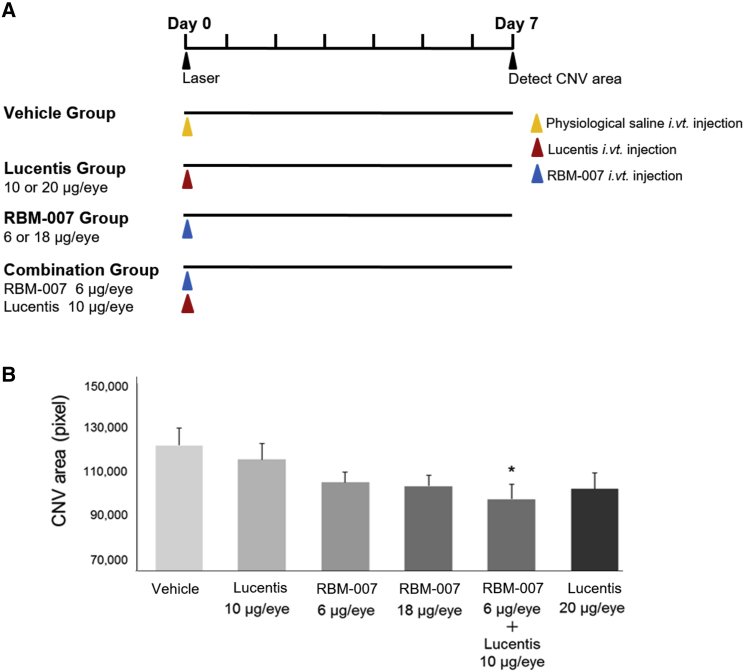
Neovascularization-Inhibitory Effect of RBM-007 in the Mouse Model of Laser-Induced CNV (A) Experimental protocol. (B) The mean group values of the neovascularization areas (pixel, mean values) were evaluated on day 7. Error bars indicate SE of n = 12 for each group. *p < 0.05, Dunnett’s test.

The CNV area in the ranibizumab groups and RBM-007 groups was decreased in a dose-dependent manner (Figure 4B). The CNV area was most suppressed in the combination group, indicating a synergistic effect of strong anti-FGF2 activity (K_D_ < 20 pM)^37^ with anti-VEGF action. Although no statistically significant difference was noted with Tukey-Kramer test among the groups, Dunnett’s test revealed a statistically significantly smaller CNV area in the RBM-007 (6 μg) + Lucentis (10 μg) group compared with the saline group (p < 0.05).

### Neovascularization-Inhibitory Effect of RBM-007 in the Rat Model of Laser-Induced CNV

**Figure 5:**
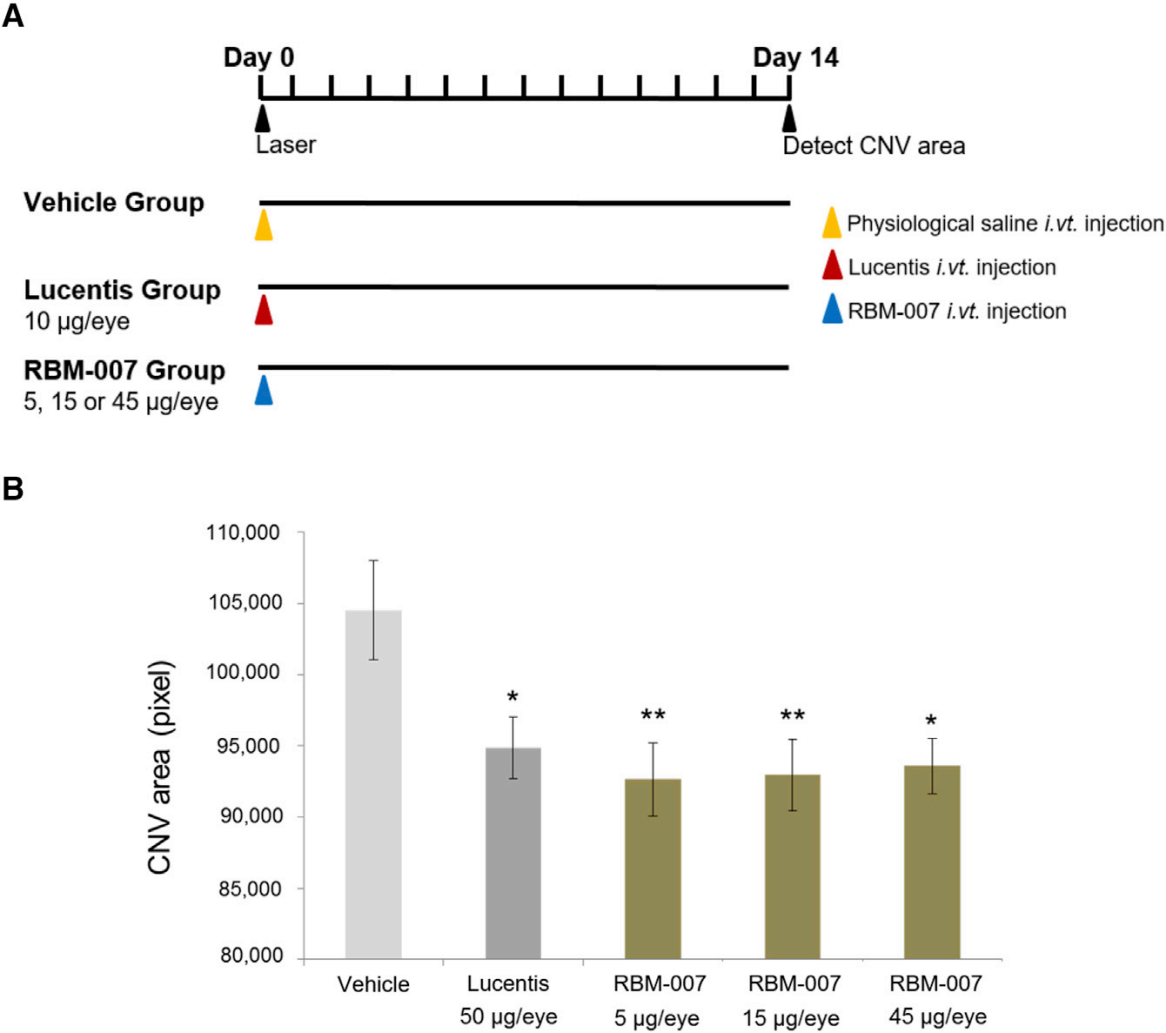
Neovascularization-Inhibitory Effect of RBM-007 in the Rat Model of Laser-Induced CNV (A) Experimental protocol. (B) The mean group values of the neovascularization areas (pixel, mean values) were evaluated on day 14 after laser irradiation. Error bars indicate SE of n = 12 for each group. *p < 0.05, **p < 0.01.

Laser-induced CNV was conducted in the eyes of the male Brown Norway (BN) rats (6 weeks, n = 12), using the same techniques as above for the mouse model. Intravitreal injection (5 μL/eye) immediately after laser induction was performed with (1) saline vehicle, (2) ranibizumab (10 μg/eye), or (3) RBM-007 (5, 15, or 45 μg/eye) (Figure 5A). After 14 days, the values of the CNV area on confocal microscopy in the ranibizumab group and all RBM-007 groups were comparable with and lower than the value in the saline group (Figure 5B). Because the p value in Bartlett’s test was 0.371 (p ˃ 0.05), indicating homogeneous variance, Dunnett’s test was conducted. The p values in Dunnett’s test were 0.0368, 0.0150, 0.009352, and 0.007337 in the Lucentis, RBM-007 high dose, RBM-007 medium dose, and RBM-007 low dose groups, respectively, compared with the controls. A CNV-inhibitory effect of RBM-007 was observed at all dose levels, although dose dependency was not confirmed.

### Subretinal Fibrosis-Inhibitory Effect of RBM-007 in the Rat Model of Laser-Induced CNV

**Figure 6:**
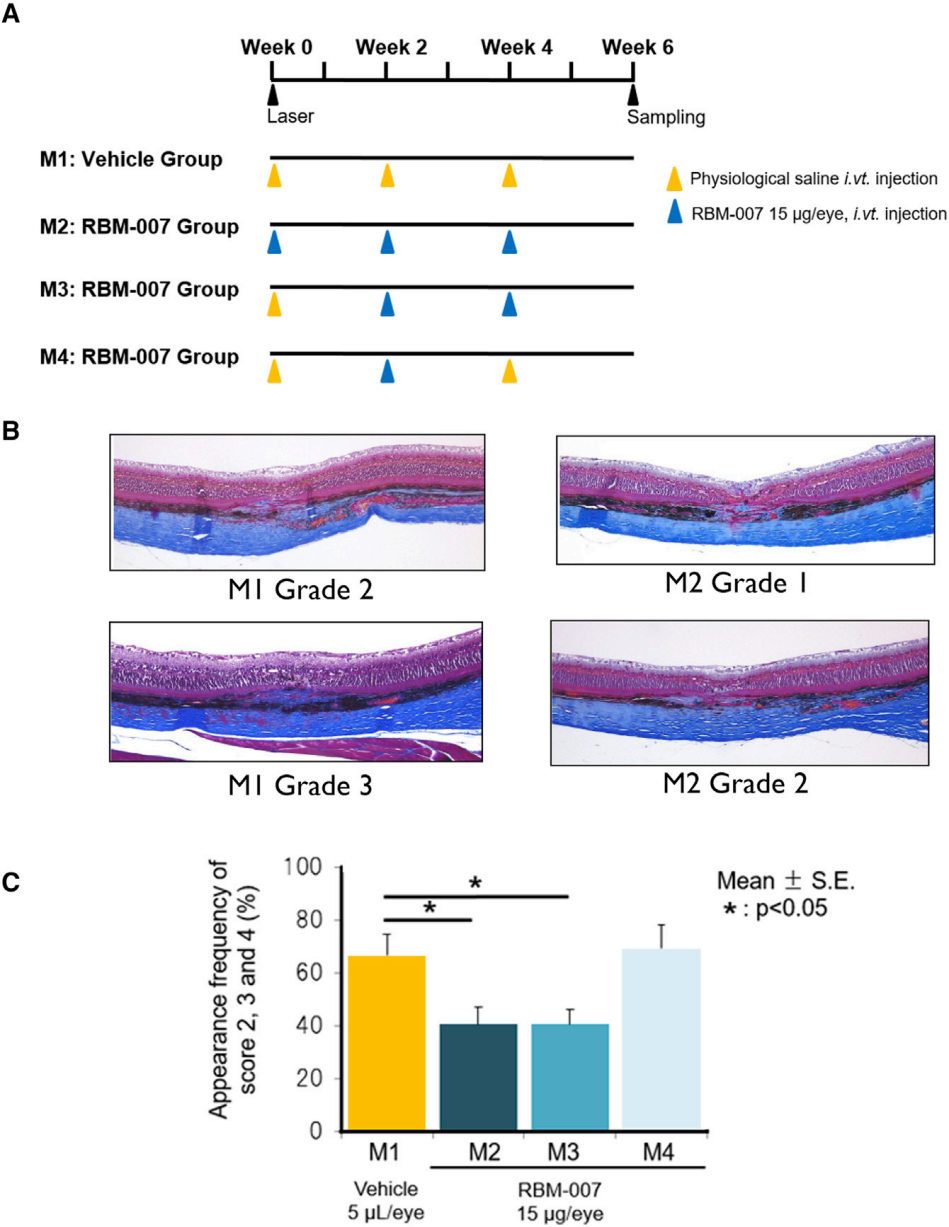
Subretinal Fibrosis-Inhibitory Effect of RBM-007 in the Rat Model of Laser-Induced CNV with Fibrosis (A) Experimental protocol and test group configuration (n = 12 for each group). (B) Representative Masson trichrome-stained sections. (C) Histopathological evaluation of whole eyes was performed 6 weeks after laser irradiation. The fibrosis at each laser injury site was graded on a scale of 0 to 4 as follows: grade 0, none; grade 1, minimal; grade 2, mild; grade 3, moderate; and grade 4, severe. The groups treated with RBM-007 for 3 injections (M2) and 2 injections (M3) had significantly lower percentages of grade 2 fibrosis or higher compared with vehicle controls (p < 0.05, Dunnett’s test).

To evaluate the fibrosis-inhibitory effect of RBM-007, *in vivo* studies were conducted in a model of laser-induced CNV and fibrosis in male BN rats (6 weeks, n = 12), using a higher power (240 mW) and longer follow-up time (6 weeks) to enhance formation of subretinal fibrosis associated with CNV. After laser irradiation, eyes received an intravitreal injection of 5 μL/eye of saline vehicle (M1 group) for comparison with RBM-007 (15 μg/eye) at intervals of 2 weeks for 3 injections (M2 group), 2 injections (M3 group), or 1 injection (M4 group) over the 6-week study period (Figure 6A). 6 weeks after laser irradiation, histopathological examination of whole eyes was conducted, and subretinal fibrosis at each laser injury site was assessed on a scale of grade 0 (no fibrosis) to grade 4 (severe fibrosis). In the M1 group, there was fibrosis at 47 of 48 sites; 16 sites had minimal, 23 sites had mild, and 8 sites had moderate grades. For the M2 group, there was fibrosis at all 49 sites; 29 sites had minimal, 15 sites had mild, and 5 sites had moderate grades. For the M3 group, there was fibrosis at all 52 sites; 31 sites had minimal, 17 sites had mild, and 4 sites had moderate grades. For the M4 group, there was fibrosis at all 48 sites; 15 sites had minimal, 24 sites had mild, and 9 sites had moderate grades. Representative Masson trichrome-stained sections are shown in Figure 6B.

Fibrosis-inhibitory effects of RBM-007 were evaluated as the percentage of laser injury sites with a score of grade 2 fibrosis or higher (Figure 6C). Compared with the saline vehicle control group (M1), groups treated with RBM-007 for 3 injections (M2) and 2 injections (M3) showed significant reductions in subretinal fibrosis (66.51% ± 8.05 SE controls versus 40.55% ± 6.54 SE (M2) and 40.42% ± 5.82 SE (M3), p < 0.05, Dunnett’s test). In contrast, the M4 group, receiving only 1 injection of RBM-007 over 6 weeks, did not have a statistically significant reduction in fibrosis scores.

### Pharmacokinetic Properties of RBM-007 in Rabbits after Intravitreal Injection

**Figure 7:**
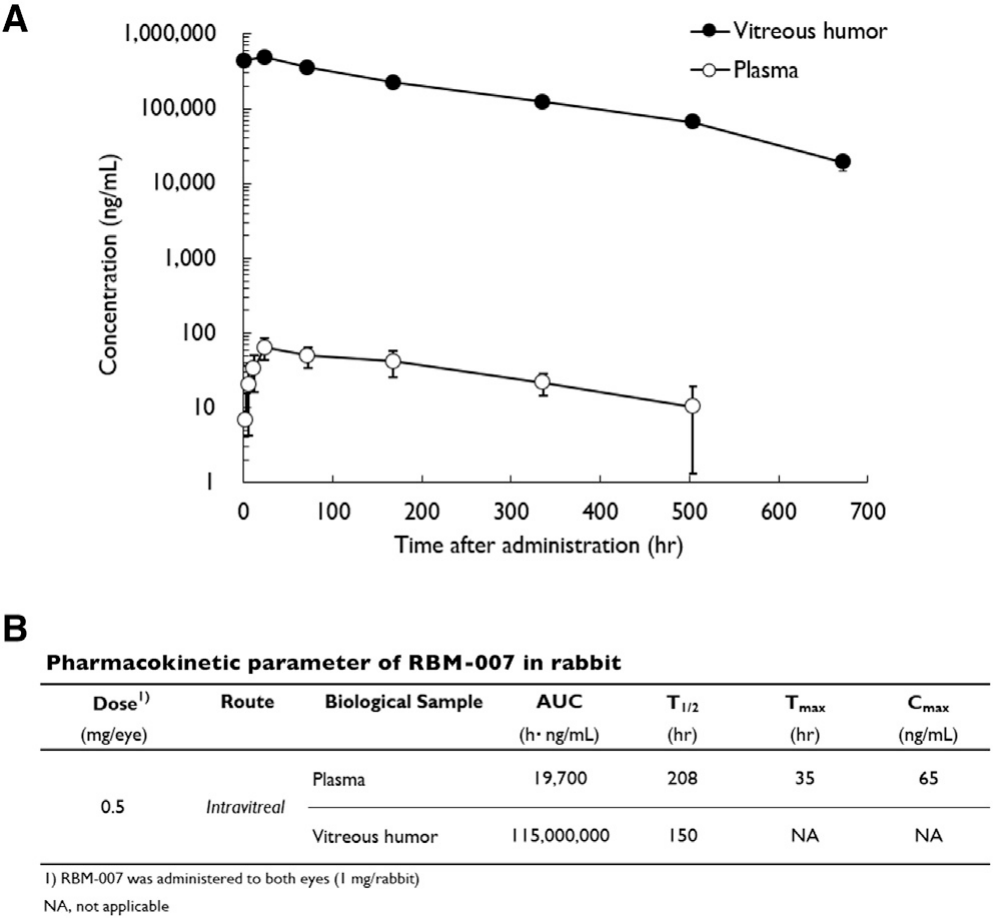
Plasma Concentrations and Vitreous Humor Concentrations of RBM-007 after Intravitreal Injection of RBM-007 (0.5 mg/Eye for 2 Eyes) to NZW Rabbits (A) Plasma and vitreous humor concentrations of RBM-007 were measured according to the indicated experimental protocol (total number of rabbits = 21, n = 3 for each time point). (B) Pharmacokinetic parameters.

The pharmacokinetic parameters of RBM-007 in both plasma and vitreous humor were determined in male rabbits after intravitreal injection at a dose of 0.5 mg/eye (1.0 mg/rabbit) with a volume of 40 μL/eye (Figure 7). The RBM-007 concentrations were determined by the ELOSA (enzyme-linked oligonucleotide sorbent assay) method. In plasma, after intravitreal injection of RBM-007, T_max_ reached 34.7 h with C_max_ of 0.065 μg/mL, and T_1/2_ was 208 h. In vitreous humor, C_0_, AUC_0–672h_, AUC_0–∞_, and T_1/2_ were 437 μg/mL, 111,000 μg·h/mL, 115,000 μg·h/mL, and 150 h, respectively. The concentration of RBM-007 in plasma was very low, less than 1/1,000, in comparison with vitreous humor concentrations of RBM-007 at all sampling times (1 h to 28 days) after intravitreal injection. On the other hand, the vitreous humor concentrations of RBM-007 were high and relatively long lasting (T_1/2_ of 150 h), showing 437 μg/mL at 1 h, 223 μg/mL at 7 days, and 123 μg/mL at 14 days. The means of C_max_ and T_max_ at 0.5 mg/eye (first and second phase) were 0.065 μg/mL and 34.7 h, respectively, and the mean of T_1/2_ at 0.5 mg/eye (second phase) was 208 h.

Based on the pharmacokinetic studies described above, it is unlikely that the systemic exposure is substantially higher than that observed in completed systemic dosing toxicity studies (data not shown). A comparison of rabbit pharmacokinetic parameters of RBM-007 with other approved nAMD therapies, such as Macugen (pegaptanib, New Drug Application [NDA] number 021756), Lucentis (ranibizumab, NDA number 125156), and Eylea (aflibercept, NDA number 125387) is provided in Table 1. The half-life of RBM-007 in vitreous humor is substantially higher than those of the approved drugs, supporting an interdose interval in future clinical studies of 1 month or greater for providing adequate efficacy. Overall, the systemic exposure from intravitreal dosing will be well below the blood levels at which toxicity is seen in animal studies, indicating a high margin of safety for potential systemic effects from intravitreal RBM-007.

**Table 1:** Comparison of Pharmacokinetic Parameters of Various Drugs in Rabbit Vitreous Humor after Intravitreal Injection

Drug	Dose (mg/Eye)	Pharmacokinetic Parameters	
		AUC_0–∞_ (μg·h/mL)	T_1/2_ (h)
RBM-007 (this study)	0.5	115,000	150
Pegaptanib sodium^56^	0.5	47,129	83
	1.18	isotope: 86,083(μg·h/g)	isotope: 152
Ranibizumab^57^	0.5	47,299	70
Aflibercept^58^	0.5	66,053	115

## Discussion

Intravitreal anti-VEGF treatments have shown dramatic visual benefits for nAMD patients.^7^, ^8^, ^9^ However, some AMD patients have incomplete responses to anti-VEGF drugs,^7^, ^8^, ^9^ and over the prolonged treatment course, persistent or recurrent disease activity can lead to poor vision outcomes.^10^, ^11^, ^12^ The reason why some nAMD patients are partial responders or nonresponders to anti-VEGF drugs is not clear,^7^, ^8^, ^9^, ^10^, ^11^, ^12^ and some factor(s) other than VEGF could be involved in nAMD progression. Submacular fibrotic scar formation is a factor associated with poor vision outcomes, and to date, no treatment is available to block retinal fibrosis.^13^

RBM-007 is a novel oligonucleotide-based aptamer with potent anti-FGF2 activity.^37^ In this study, we demonstrated that excess FGF2 plays a key role in nAMD by stimulating angiogenesis and that RBM-007 blocks both CNV and subretinal fibrosis. Moreover, combined intravitreal injections of RBM-007 and ranibizumab (Lucentis) showed synergistic action in preventing CNV.

Although FGF2 is characterized by stimulating fibroblast proliferation, the role of FGF2 in fibrotic scar formation in the retina has not been investigated. In this study, we showed that FGF2 alone does not induce EMT in RPE cells but greatly stimulates TGF-β2-induced EMT. Therefore, one could assume that TGF-β2 and FGF2 combination treatment results in more EMT than TGF-β2 alone and that the main part of the EMT phenotype could revert to TGF-β2 alone by inhibiting FGF2 with RBM-007. These *in vitro* findings are consistent with the *in vivo* results regarding the subretinal fibrosis-inhibitory effect of RBM-007 in the rat model of laser-induced CNV (Figure 6).

Although the mechanism behind this cooperative action of TGF-β2 and FGF2 remains to be investigated, it is worth mentioning the report by Strutz et al.^31^ They noticed that EMT is induced efficiently in cultured proximal tubular epithelium by coincubation of TGF-β1 and FGF2. They speculated that one contribution of FGF2 to EMT is stimulation of microenvironmental proteases; namely, that loss of integrity of the basement membrane is an important part of EMT and that FGF2 can facilitate this process by stimulating secretion of matrix metalloproteinases.

Although several studies have suggested FGF2 as a therapeutic target in diseases such as rheumatoid arthritis and other bone diseases,^40^ and a number of anti-FGF2 monoclonal antibodies (mAbs) have been developed previously to neutralize various activities of FGF2 *in vitro* and, in some cases, *in vivo*,^41^, ^42^, ^43^ FGF2 has not been developed for retinal angiogenic eye disease.

A few case reports describe treating AMD and other retina disorders with dobesilate, which has long been used clinically as an FGF inhibitor.^44^, ^45^, ^46^, ^47^, ^48^, ^49^ Dobesilate is a general inhibitor of FGF signaling and not specific to FGF2. However, given that FGF2 is a major FGF protein expressed in CNV membranes, it is reasonable to speculate that the therapeutic effect of intravitreal dobesilate could be due to blockade of FGF2-induced signaling toward CNV.

The effectiveness of RBM-007 in nAMD therapy can be further supported by findings that VEGF and FGF2 are colocalized in epiretinal and CNV membranes^21^, ^50^, ^51^ and that FGF receptor double-conditional knockout (*Fgfr1/2*) mice show a marked reduction in CNV and a decrease in FGF2 level upon laser injury.^52^

We have completed a comprehensive nonclinical program to evaluate the pharmacology, pharmacokinetics, and toxicity of RBM-007, some of which are presented in this article. It was observed that, after intravitreal RBM-007 injection, plasma levels were less than 1/1,000 in comparison with vitreous humor concentrations, suggesting low systemic exposure. Also, no significant ocular toxicity was seen in rabbits and monkeys upon intravitreal injections (1 mg/eye) of RBM-007 (data not shown). The vitreous humor concentrations of RBM-007 were high and relatively long lasting (T_1/2_ of 150 h) in comparison with available anti-VEGF drugs (Table 1), which offers potential for extended therapy duration that could be beneficial for patients who, in real-world clinical management, receive less frequent intravitreal injections. Based on these preclinical data, a clinical trial program has been initiated to assess the safety, tolerability, and efficacy of intravitreal RBM-007 in patients with active nAMD.^53^

## Materials and Methods

### Materials

RBM-007 used for *in vitro* (cell) and *in vivo* (animal) experiments was 5′- and 3′-conjugated with 40-kDa PEG (SUNBRIGHT GL2-400TS, NOF) and an inverted dT (idT), respectively, were prepared by chemical synthesis (Gene Design and Nitto Denko Avecia).^33^ Other protein factors used were recombinant human FGF2 (Lonza), human TGF-β2 (PeproTech) and ranibizumab (Lucentis, Novartis). Human RPE cells were purchased from Lonza.

### Animals

Female C57BL/6J mice were obtained from Charles River Laboratories Japan. Male BN rats were obtained from Japan SLC, Japan. Male New Zealand White (NZW) rabbits were obtained from Kitayama Labes, Japan. Mice, rats, and rabbits were maintained under special pathogen-free conditions. Animal experiments were performed in accordance with the Guidelines for Animal Experiments of the Institute of Medical Science, University of Tokyo (Japan); Life Science Laboratories; and Shin Nippon Biomedical Laboratories. The experimental protocols for the mouse Matrigel plug assay were approved by the animal experiment committee of the Institute of Medical Science, University of Tokyo, and the protocol for mouse and rat CNV experiments was approved by the animal experiment and licensing committee of Life Science Laboratories. The protocol for pharmacokinetic study of rabbit intravitreal injections was approved by the animal experiment and licensing committee of Shin Nippon Biomedical Laboratories.

### EMT Assay of RPE Cells

RPE cells cultured at 37°C were seeded at 1 × 10^5^ cells/well into 96-well microtiter culture plates. After incubation for 24 h, cells were treated with FGF2 (2 ng/mL) and TGF-β2 (3 ng/mL) with and without RBM-007 (10 and 100 ng/mL) for 3 days. The mRNA expression levels of the EMT biomarkers α-SMA and collagen type I were evaluated with quantitative real-time PCR.

### Matrigel Plug Assay

0.5 mL Matrigel Matrix GFR (growth factor reduced)-PRF (phenol red free) (BD Biosciences) solution was mixed with 1 μg FGF2 and subcutaneously implanted into the right flank of female C57BL/6J mice (8 weeks, n = 3). RBM-007 was administered intraperitoneally every day at doses of 1, 3, and 10 mg/kg, and Matrigel plugs were removed and photographed on day 7 post-implantation. Grading of angiogenesis was determined by the hemoglobin concentration in the Matrigel plug, using the cyanmethemoglobin method according to the manufacturer’s instructions. In brief, the Matrigel plug was macerated with an equal volume of red blood cell (RBC) lysis solution (Sigma-Aldrich) and incubated overnight on ice. The preparation samples were added to Drabkin’s solution (Sigma-Aldrich) containing 0.3% Brij-35 and incubated for 30 min at room temperature. The optical density at 540 nm (OD_540_) values of the samples were measured in a microplate reader, and the hemoglobin concentrations were calculated in accordance with hemoglobin standards (Sigma-Aldrich).

### Mouse Laser-Induced CNV Models

Mydrin-P ophthalmic solution was instilled into the eyes of the male C57BL/6J mice (6 weeks, n = 8∼10 for each dose group) to dilate the pupils. Then laser irradiation (wavelength, 532 nm; spot size, 50 μm; irradiation time, 0.1 s; laser output, 120 mW) was conducted on 6 sites of the eye using a slit lamp (SL-130) and a multicolor laser photocoagulator (MC-300), avoiding large retinal capillaries.^54^ Immediately after CNV induction by laser irradiation, the test and control substances were administered via intravitreal injection, using microsyringes and 33G needles, at 2 μL/eye for the mouse model: (1) saline vehicle solution, (2) ranibizumab (10 or 20 μg/eye, Lucentis, Genentech), RBM-007 (6 or 18 μg/eye), and a combination of ranibizumab (10 μg/eye) and RBM-007 (6 μg/eye) (Figure 4A). Seven days after laser irradiation, a 4% FITC-dextran solution was administered into the tail vein at a volume of 0.5 ml/animal. One to five minutes after administration of FITC-dextran, the animals were euthanized by cervical dislocation. The eyeballs were removed and fixed in 4% paraformaldehyde-phosphate buffer for 12–24 h. Choroidal flat mounts were prepared under a stereoscopic microscope. Photographs of CNV sites were taken using a confocal microscope. Throughout the laser-induced CNV animal experiments, successful irradiation was confirmed for each irradiation as air bubble formation in the eye of animal models.

### Rat Laser-Induced CNV and Subretinal Fibrosis Models

The same technique was employed in the rat model using male BN rats (6 weeks, n = 12). For the CNV model, laser settings were spot size 80 μm and irradiation time of 0.05 s at 120 mW at 8 sites in the retina-choroid. For the subretinal fibrosis CNV model, the laser power used was 240 mW. Immediately after laser irradiation, intravitreal injection was performed (5 μL/eye). For the CNV studies, (1) saline vehicle, (2) ranibizumab (10 μg/eye), and (3) RBM-007 (5, 15, or 45 μg/eye) were used (Figure 5A). For the subretinal fibrosis CNV studies, (1) saline vehicle and (2) RBM-007 (15 μg/eye for a single injection or 2 or 3 injections at 2-week intervals) were used (Figure 6A). After laser irradiation, for the CNV studies, the FITC-dextran protocol as above was used 14 days after laser irradiation, with preparation of enucleated eyes for confocal microscopy of flat mount choroid. For the subretinal fibrosis studies, after 6 weeks, animals were sacrificed, and the enucleated eyes were prepared for histopathologic studies to quantify subretinal fibrosis using light microscopy. The eyes were embedded in paraffin blocks and sectioned and stained with Masson trichrome according to routine methods. Fibrosis was graded in a blinded manner by two or three independent investigators according to the following scale: grade 0, none; grade 1, minimal; grade 2, mild; grade 3, moderate; grade 4, severe.

### ELOSA

The plasma and vitreous humor levels of RBM-007 were measured using an ELOSA according to a method reported previously.^55^ The RNA sequences of the detection probe and capture probe were designed based on homologous regions. The reliability of the ELOSA methods for quantification of RBM-007 concentrations in rabbit plasma and vitreous humor was validated beforehand. The pharmacokinetic (PK) parameters were derived by using noncompartmental models with Phoenix WinNonlin version 6.3 (Pharsight, Mountain View, CA, USA).

### Pharmacokinetics Analysis

RBM-007 was administered intravitreally to NZW rabbits (Kitayama Labes, males aged 25 weeks) at 0.5 mg/eye (1.0 mg/both eyes), and plasma and vitreous humor of both eye were collected 1, 24, 72, 168, 336, 504, and 672 h after administration. Three animals were analyzed at each time point. The RBM-007 concentration in plasma and vitreous humor was measured with the ELOSA method.

### Statistical Analysis

All data regarding *in vivo* experiments show mean and SE calculated by Microsoft Excel. The clinical scores and histopathological scores were analyzed using Ekuseru-Toukei 2006 for Windows (Social Survey Research Information). Differences between vehicle and treatment groups were considered statistically significant at p < 0.05.

## Author Contributions

Y.M. performed *in vitro* cell studies. Y.M., Y. Nonaka, S.F., and M.F. contributed to the *in vivo* animal experiments. K.A. contributed to the oligonucleotide analysis. T.N., Y.A., and R.B.B. contributed to evaluation of the data. M.F. and Y. Nakamura planned the study. Y. Nakamura coordinated the project and wrote the manuscript. R.B.B. edited the manuscript.

## Conflicts of Interest

All authors except for R.B.B. are employees and shareholders of RIBOMIC, Inc.

## Supplemental Information

Document S1. Figures S1–S3

Document S2. Article plus Supplemental Information

## References

[1] Kashani A.H. (2016). Stem cell therapy in nonneovascular age-related macular degeneration. Invest. Ophthalmol. Vis. Sci..

[2] Kashani A.H., Lebkowski J.S., Rahhal F.M., Avery R.L., Salehi-Had H., Dang W., Lin C.M., Mitra D., Zhu D., Thomas B.B. (2018). A bioengineered retinal pigment epithelial monolayer for advanced, dry age-related macular degeneration. Sci. Transl. Med..

[3] Rudnicka A.R., Kapetanakis V.V., Jarrar Z., Wathern A.K., Wormald R., Fletcher A.E., Cook D.G., Owen C.G. (2015). Incidence of late-stage age-related macular degeneration in American whites: systematic review and meta-analysis. Am. J. Ophthalmol..

[4] Vanderbeek B.L., Zacks D.N., Talwar N., Nan B., Musch D.C., Stein J.D. (2011). Racial differences in age-related macular degeneration rates in the United States: a longitudinal analysis of a managed care network. Am. J. Ophthalmol..

[5] Blaauwgeers H.G., Holtkamp G.M., Rutten H., Witmer A.N., Koolwijk P., Partanen T.A., Alitalo K., Kroon M.E., Kijlstra A., van Hinsbergh V.W., Schlingemann R.O. (1999). Polarized vascular endothelial growth factor secretion by human retinal pigment epithelium and localization of vascular endothelial growth factor receptors on the inner choriocapillaris. Evidence for a trophic paracrine relation. Am. J. Pathol..

[6] Drolet D.W., Green L.S., Gold L., Janjic N. (2016). Fit for the eye: aptamers in ocular disorders. Nucleic Acid Ther..

[7] Brown D.M., Kaiser P.K., Michels M., Soubrane G., Heier J.S., Kim R.Y., Sy J.P., Schneider S., ANCHOR Study Group (2006). Ranibizumab versus verteporfin for neovascular age-related macular degeneration. N. Engl. J. Med..

[8] Heier J.S., Brown D.M., Chong V., Korobelnik J.F., Kaiser P.K., Nguyen Q.D., Kirchhof B., Ho A., Ogura Y., Yancopoulos G.D., VIEW 1 and VIEW 2 Study Groups (2012). Intravitreal aflibercept (VEGF trap-eye) in wet age-related macular degeneration. Ophthalmology.

[9] Martin D.F., Maguire M.G., Fine S.L., Ying G.S., Jaffe G.J., Grunwald J.E., Toth C., Redford M., Ferris F.L., Comparison of Age-related Macular Degeneration Treatments Trials (CATT) Research Group (2012). Ranibizumab and bevacizumab for treatment of neovascular age-related macular degeneration: two-year results. Ophthalmology.

[10] Rofagha S., Bhisitkul R.B., Boyer D.S., Sadda S.R., Zhang K., SEVEN-UP Study Group (2013). Seven-year outcomes in ranibizumab-treated patients in ANCHOR, MARINA, and HORIZON: a multicenter cohort study (SEVEN-UP). Ophthalmology.

[11] Bhisitkul R.B., Desai S.J., Boyer D.S., Sadda S.R., Zhang K. (2016). Fellow eye comparisons for 7-year outcomes in ranibizumab-treated AMD subjects from ANCHOR, MARINA, and HORIZON (SEVEN-UP Study). Ophthalmology.

[12] Maguire M.G., Martin D.F., Ying G.S., Jaffe G.J., Daniel E., Grunwald J.E., Toth C.A., Ferris F.L., Fine S.L., Comparison of Age-related Macular Degeneration Treatments Trials (CATT) Research Group (2016). Five-year outcomes with anti-vascular endothelial growth factor treatment of neovascular age-related macular degeneration: the comparison of age-related macular degeneration treatments trials. Ophthalmology.

[13] Daniel E., Toth C.A., Grunwald J.E., Jaffe G.J., Martin D.F., Fine S.L., Huang J., Ying G.S., Hagstrom S.A., Winter K., Maguire M.G., Comparison of Age-related Macular Degeneration Treatments Trials Research Group (2014). Risk of scar in the comparison of age-related macular degeneration treatments trials. Ophthalmology.

[14] Friedlander M. (2007). Fibrosis and diseases of the eye. J. Clin. Invest..

[15] Jaffe G.J., Martin D.F., Toth C.A., Daniel E., Maguire M.G., Ying G.S., Grunwald J.E., Huang J., Comparison of Age-related Macular Degeneration Treatments Trials Research Group (2013). Macular morphology and visual acuity in the comparison of age-related macular degeneration treatments trials. Ophthalmology.

[16] Bressler N.M., Frost L.A., Bressler S.B., Murphy R.P., Fine S.L. (1988). Natural course of poorly defined choroidal neovascularization associated with macular degeneration. Arch. Ophthalmol..

[17] Wong T.Y., Chakravarthy U., Klein R., Mitchell P., Zlateva G., Buggage R., Fahrbach K., Probst C., Sledge I. (2008). The natural history and prognosis of neovascular age-related macular degeneration: a systematic review of the literature and meta-analysis. Ophthalmology.

[18] Pauleikhoff D. (2005). neovascular age-related macular degeneration: Natural History and Treatment Outcomes. Retina.

[19] Sivaprasad S., Saleh G.M., Jackson H. (2006). Does lesion size determine the success rate of photodynamic therapy for age-related macular degeneration?. Eye (Lond.).

[20] Cohen S.Y., Oubraham H., Uzzan J., Dubois L., Tadayoni R. (2012). Causes of unsuccessful ranibizumab treatment in exudative age-related macular degeneration in clinical settings. Retina.

[21] Schultz G.S., Grant M.B. (1991). Neovascular growth factors. Eye (Lond.).

[22] Vinding T. (1990). Occurrence of drusen, pigmentary changes and exudative changes in the macula with reference to age-related macular degeneration. An epidemiological study of 1000 aged individuals. Acta Ophthalmol. (Copenh.).

[23] Bikfalvi A., Klein S., Pintucci G., Rifkin D.B. (1997). Biological roles of fibroblast growth factor-2. Endocr. Rev..

[24] Presta M., Dell’Era P., Mitola S., Moroni E., Ronca R., Rusnati M. (2005). Fibroblast growth factor/fibroblast growth factor receptor system in angiogenesis. Cytokine Growth Factor Rev..

[25] Ferrara N., Gerber H.P., LeCouter J. (2003). The biology of VEGF and its receptors. Nat. Med..

[26] Tomanek R.J., Sandra A., Zheng W., Brock T., Bjercke R.J., Holifield J.S. (2001). Vascular endothelial growth factor and basic fibroblast growth factor differentially modulate early postnatal coronary angiogenesis. Circ. Res..

[27] Belgore F., Lip G.Y.H., Blann A.D. (2003). Basic fibroblast growth factor induces the secretion of vascular endothelial growth factor by human aortic smooth muscle cells but not by endothelial cells. Eur. J. Clin. Invest..

[28] Malabanan K.P., Kanellakis P., Bobik A., Khachigian L.M. (2008). Activation transcription factor-4 induced by fibroblast growth factor-2 regulates vascular endothelial growth factor-A transcription in vascular smooth muscle cells and mediates intimal thickening in rat arteries following balloon injury. Circ. Res..

[29] Cao R., Bråkenhielm E., Pawliuk R., Wariaro D., Post M.J., Wahlberg E., Leboulch P., Cao Y. (2003). Angiogenic synergism, vascular stability and improvement of hind-limb ischemia by a combination of PDGF-BB and FGF-2. Nat. Med..

[30] Birsner A.E., Benny O., D’Amato R.J. (2014). The corneal micropocket assay: a model of angiogenesis in the mouse eye. J. Vis. Exp..

[31] Strutz F., Zeisberg M., Ziyadeh F.N., Yang C.Q., Kalluri R., Müller G.A., Neilson E.G. (2002). Role of basic fibroblast growth factor-2 in epithelial-mesenchymal transformation. Kidney Int..

[32] Chen J., Chen G., Yan Z., Guo Y., Yu M., Feng L., Jiang Z., Guo W., Tian W. (2014). TGF-β1 and FGF2 stimulate the epithelial-mesenchymal transition of HERS cells through a MEK-dependent mechanism. J. Cell. Physiol..

[33] Cordeiro M.F., Reichel M.B., Gay J.A., D’Esposita F., Alexander R.A., Khaw P.T. (1999). Transforming growth factor-beta1, -beta2, and -beta3 in vivo: effects on normal and mitomycin C-modulated conjunctival scarring. Invest. Ophthalmol. Vis. Sci..

[34] Nassar K., Lüke J., Lüke M., Kamal M., Abd El-Nabi E., Soliman M., Rohrbach M., Grisanti S. (2011). The novel use of decorin in prevention of the development of proliferative vitreoretinopathy (PVR). Graefes Arch. Clin. Exp. Ophthalmol..

[35] Cordeiro M.F., Mead A., Ali R.R., Alexander R.A., Murray S., Chen C., York-Defalco C., Dean N.M., Schultz G.S., Khaw P.T. (2003). Novel antisense oligonucleotides targeting TGF-beta inhibit in vivo scarring and improve surgical outcome. Gene Ther..

[36] Ishikawa K., Yoshida S., Nakao S., Nakama T., Kita T., Asato R., Sassa Y., Arita R., Miyazaki M., Enaida H. (2014). Periostin promotes the generation of fibrous membranes in proliferative vitreoretinopathy. FASEB J..

[37] Jin L., Nonaka Y., Miyakawa S., Fujiwara M., Nakamura Y. (2016). Dual therapeutic action of a neutralizing anti-FGF2 aptamer in bone disease and bone cancer pain. Mol. Ther..

[38] Ellington A.D., Szostak J.W. (1990). In vitro selection of RNA molecules that bind specific ligands. Nature.

[39] Tuerk C., Gold L. (1990). Systematic evolution of ligands by exponential enrichment: RNA ligands to bacteriophage T4 DNA polymerase. Science.

[40] Yamashita A., Yonemitsu Y., Okano S., Nakagawa K., Nakashima Y., Irisa T., Iwamoto Y., Nagai Y., Hasegawa M., Sueishi K. (2002). Fibroblast growth factor-2 determines severity of joint disease in adjuvant-induced arthritis in rats. J. Immunol..

[41] Matsuzaki K., Yoshitake Y., Matuo Y., Sasaki H., Nishikawa K. (1989). Monoclonal antibodies against heparin-binding growth factor II/basic fibroblast growth factor that block its biological activity: invalidity of the antibodies for tumor angiogenesis. Proc. Natl. Acad. Sci. USA.

[42] Rege A.A., Bjercke R.J., Erichsen D., Owens R., Stephan C.C., Brock T.A. (1999). Development of novel monoclonal antibodies for the analysis of functional sites in FGF-2. Growth Factors.

[43] Wang L., Park H., Chhim S., Ding Y., Jiang W., Queen C., Kim K.J. (2012). A novel monoclonal antibody to fibroblast growth factor 2 effectively inhibits growth of hepatocellular carcinoma xenografts. Mol. Cancer Ther..

[44] Cuevas P., Outeiriño L., Azanza C., Giménez-Gallego G. (2012). Intravitreal dobesilate in the treatment of choroidal neovascularisation associated with age-related macular degeneration: report of two cases. BMJ Case Rep..

[45] Cuevas P., Outeiriño L.A., Angulo J., Giménez-Gallego G. (2012). Treatment of Stargardt disease with dobesilate. BMJ Case Rep..

[46] Cuevas P., Outeiriño L.A., Angulo J., Giménez-Gallego G. (2012). Chronic cystoid macular oedema treated with intravitreal dobesilate. BMJ Case Rep..

[47] Cuevas P., Outeiriño L.A., Angulo J., Giménez-Gallego G. (2012). Treatment of dry age-related macular degeneration with dobesilate. BMJ Case Rep..

[48] Cuevas P., Outeiriño L.A., Azanza C., Angulo J., Giménez-Gallego G. (2012). Short-term efficacy of intravitreal dobesilate in central serous chorioretinopathy. Eur. J. Med. Res..

[49] Cuevas P., Outeiriño L.A., Azanza C., Angulo J., Giménez-Gallego G. (2013). Dobesilate for dry age-related macular degeneration. J. Biomed. Sci. Eng..

[50] Amin R., Puklin J.E., Frank R.N. (1994). Growth factor localization in choroidal neovascular membranes of age-related macular degeneration. Invest. Ophthalmol. Vis. Sci..

[51] Frank R.N., Amin R.H., Eliott D., Puklin J.E., Abrams G.W. (1996). Basic fibroblast growth factor and vascular endothelial growth factor are present in epiretinal and choroidal neovascular membranes. Am. J. Ophthalmol..

[52] Oladipupo S.S., Smith C., Santeford A., Park C., Sene A., Wiley L.A., Osei-Owusu P., Hsu J., Zapata N., Liu F. (2014). Endothelial cell FGF signaling is required for injury response but not for vascular homeostasis. Proc. Natl. Acad. Sci. USA.

[53] ClinicalTrials.gov. RBM-007 in Subjects with Exudative Age-related Macular Degeneration (SUSHI). https://clinicaltrials.gov/ct2/show/NCT03633084.

[54] Campa C., Kasman I., Ye W., Lee W.P., Fuh G., Ferrara N. (2008). Effects of an anti-VEGF-A monoclonal antibody on laser-induced choroidal neovascularization in mice: optimizing methods to quantify vascular changes. Invest. Ophthalmol. Vis. Sci..

[55] Healy J.M., Lewis S.D., Kurz M., Boomer R.M., Thompson K.M., Wilson C., McCauley T.G. (2004). Pharmacokinetics and biodistribution of novel aptamer compositions. Pharm. Res..

[56] U.S. Food and Drug Administration. Drug Approval Package: Macugen (Pegaptanib Sodium) Injection.https://www.accessdata.fda.gov/drugsatfda_docs/nda/2004/21-756_Macugen.cfm.

[57] U.S. Food and Drug Administration. Drug Approval Package: Lucentis (Ranibizumab) Injection. https://www.accessdata.fda.gov/drugsatfda_docs/nda/2006/125156s0000_LucentisTOC.cfm.

[58] U.S. Food and Drug Administration. Drug Approval Package: Eylea (Aflibercept) Injection. https://www.accessdata.fda.gov/drugsatfda_docs/nda/2011/125387s0000TOC.cfm.

