# Supplementary material for: Anti-Angiogenic and Anti-Scarring Dual Action of an Anti-Fibroblast Growth Factor 2 Aptamer in Animal Models of Retinal Disease
Source: Mol Ther Nucleic Acids. 2019 Aug 1;17:819–28. doi: 10.1016/j.omtn.2019.07.018 (PMC6716068; doi:10.1016/j.omtn.2019.07.018)
Supplement: Document S1. Figures S1–S3 [file mmc1.pdf]

OMTN, Volume *17*

Supplemental Information

Anti-Angiogenic and Anti-Scarring Dual Action of an Anti-Fibroblast Growth Factor 2 Aptamer in Animal Models of Retinal Disease

Yusaku Matsuda, Yosuke Nonaka, Satoshi Futakawa, Hirotaka Imai, Kazumasa Akita, Toshiaki Nishihata, Masatoshi Fujiwara, Yusuf Ali, Robert B. Bhisitkul, and Yoshikazu Nakamura

SUPPLEMENTARY INFORMATION

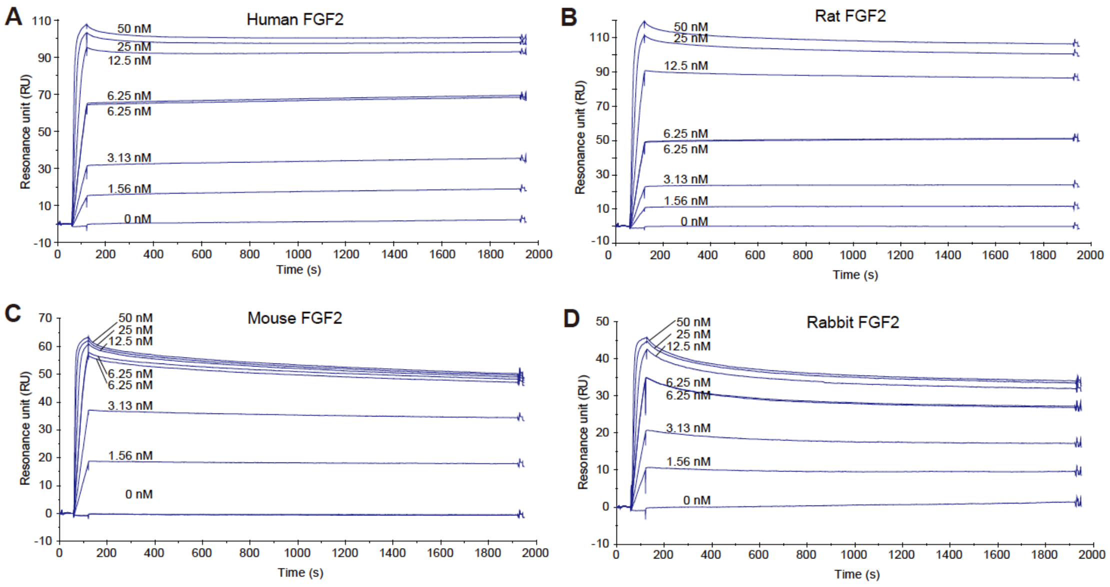

E

	K_a (1/Ms)	K_d (1/s)	Rmax (RU)	K_D (M)	Chi^2 (RU^2)
Human FGF2	7.849e6	1.649e-5	98.85	2.101e-12	4.12
Rat FGF2	7.606e6	4.163e-5	109.7	5.474e-12	3.69
Mouse FGF2	1.663e7	1.157e-4	58.42	6.955e-12	0.551
Rabbit FGF2	6.586e6	1.780e-4	40.51	2.702e-11	0.787

Figure S1. Binding profile of RBM-007 oligonucleotide to various animal FGF2 proteins measured by SPR

5'-biotine labeled RBM-007 oligonucleotide was immobilized on a streptavidin-sensor chip and different concentrations of FGF2 proteins were injected as described previously.[37] Experimental conditions and procedures are the same as in Materials and Methods. Shown are SPR sensorgrams monitoring the affinity of RBM-007 oligonucleotide to FGF2 proteins from human (A), rat (B), mouse (C), and rabbit (D). (E) Estimated parameters including k_a (association rate), k_d (dissociation rate) and K_D (dissociation constant) by using BIAevaluation 3.0 software.

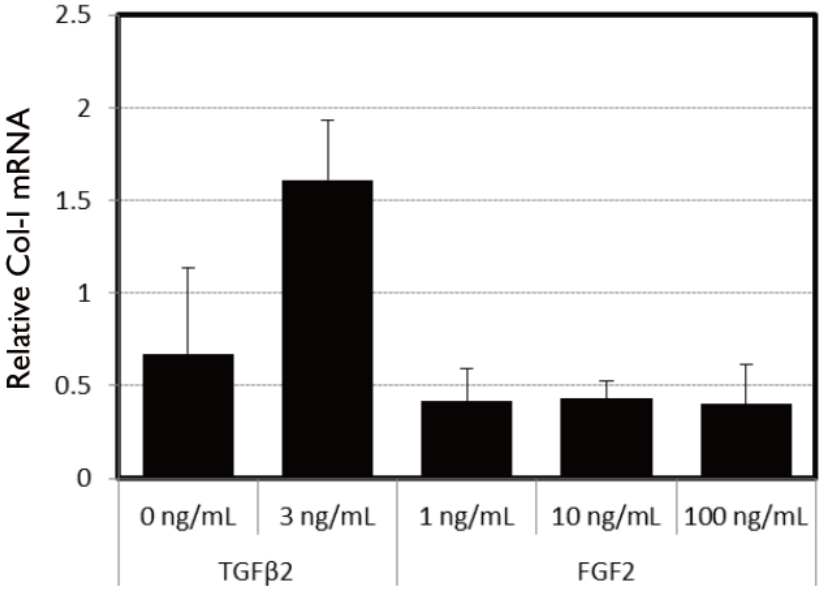

Figure S2. Effects of TGFβ2 and FGF2 on EMT in RPE cells

The mRNA level of collagen type I was examined by qRT-PCR amplification in RPE cells after addition of TGFβ2 and FGF2. Experimental conditions and procedures are the same as in Materials and Methods.

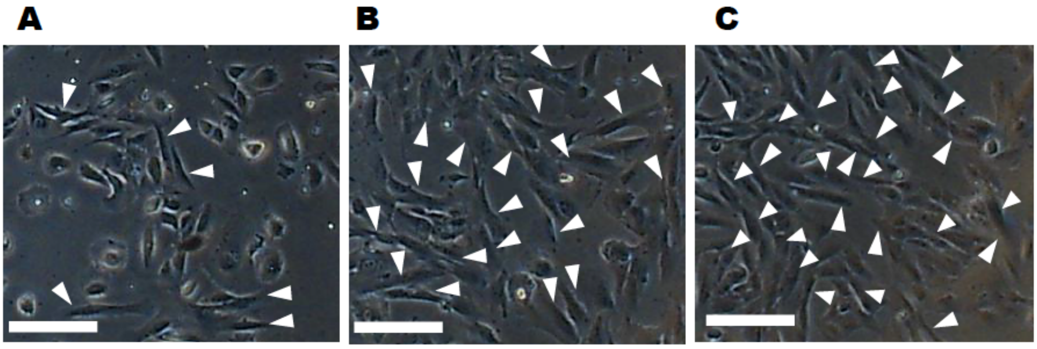

Figure S3. Morphological changes of retinal pigment epithelium (RPE) cells in response to FGF2 plus TGFβ2 stimulation

Human primary RPE cells purchased from Lonza Japan were cultured in RtEBM medium containing Lonza’s supplements in accordance with Lonza's protocol. Three passaged RPE cells were used for this experiment. RPE cells cultured in 24-well culture plate at a concentration of 2×10^4 cells per well were incubated at 37°C, 5% CO_2 for 1 day. After incubation, culture medium was removed and replaced with fresh medium containing (A) no addition, (B) TGFβ2 (3 ng/mL) or (C) TGFβ2 (3 ng/mL) plus FGF2 (0.074 ng/mL). 2 days cultivated cells were qualitatively evaluated the morphological change by microscopic observation. White arrow shows spindle-shaped (fibroblast like) cells. Scale bars represent 100 μm. Note that the passaged RPE cells were slightly contaminated with spindle-shaped cells.
